# The Femoral Vein as a Long-Term Aorto-Iliac Graft for Aortic Infection and Aortitis

**DOI:** 10.1007/s00268-022-06460-w

**Published:** 2022-02-03

**Authors:** W. Eilenberg, J. Klopf, C. M. Domenig, M. Klinger, F. Wolf, B. Gollackner, J. Nanobachvili, C. Neumayer

**Affiliations:** 1 Department of General Surgery, Division of Vascular Surgery, Medical University of Vienna, General Hospital of Vienna, Waehringer Guertel 18-20, 1090 Vienna, Austria; 2 Department of Biomedical Imaging and Image Guided Therapy, Division of Cardiovascular and Interventional Radiology, Medical University of Vienna, General Hospital of Vienna, Waehringer Guertel 18-20, 1090 Vienna, Austria

## Abstract

**Background:**

Reconstruction of the aorto-iliac segment with femoral vein (FV) as substitute for infected synthetic grafts or mycotic aneurysms constitutes the most sustainably convenient alternative. The aim of this study was to evaluate the long-term outcome of up to 16 years of follow-up, analysing the morphologic adaption of the FV with special emphasis on the distal and proximal anastomoses.

**Methods:**

We conducted a retrospective study of 22 patients with 109 computed tomography angiograms (CTAs) treated between August 2001 and January 2020 in case of aortic infection/aortitis. Morphologic changes like anastomotic dilatation/stenosis as well as changes of FV wall thickness were retrospectively analysed in pre- and postoperative CTAs.

**Results:**

Elective procedure was done in 17/22 (77%) cases, and 5/22 (23%) patients required emergent surgery. The median follow-up was 91.5 months (P_25_;P_75_ = 21;117). Cross-sectional diameter of proximal (20.38 ± 3.77 vs 22.04 ± 3.97 mm, *p* = 0.007) and distal anastomoses (13.05 ± 4.23 vs 14.61 ± 5.19 mm, *p* = 0.05) increased significantly, as well as the proximal and distal anastomotic areas (3.36 ± 1.29 vs 4.32 ± 1.63 mm^2^, *p* = 0.04 and 0.99 ± 0.48 vs 1.25 ± 0.72 mm^2^, *p* = 0.023, respectively). Venous wall thickness was significantly reduced at the anastomotic site (1.74 ± 0.46 vs 1.24 ± 0.31 mm, *p* = 0.001). The upper thigh diameter did not differ before and after harvesting of the FV (161.6 ± 29.1 vs. 178.2 ± 23.3 mm, *p* = 0.326, respectively).

**Conclusion:**

This long-term CTA follow-up study showed that the FV wall becomes thinner at the anastomotic site, and the anastomoses dilate with time without rupture. The FV is a durable conductor after replacement of the aorto-iliac segment due to aortic infection. Further CTA studies from more centres are warranted to evaluate the risk of vein rupture.

## Introduction

Reconstruction of the aorto-iliac segment with autologous femoral vein (FV) is one of several techniques for the treatment of mycotic aortic aneurysm (mAAA) as well as for replacement of infected prosthetic grafts. This technique yields a good infection control and acceptable long-term survival [1–4]. Although these infections are rare, they ultimately lead to life-threatening conditions including sepsis, multiple organ failure, rupture or finally to the death of the patient. Antimicrobial therapy may be an option for high-risk patients only, although the complete eradication of the infection is hardly possible, even by wide debridement and complete removal of the infected graft [5, 6]. Other surgical options after removal of infected prosthesis include: the extra-anatomic axillo-femoral bypass, cryopreserved aortic allografts and biological prosthesis, although their use is related to complications like secondary rupture, reduced long-term patency, aneurysmal degeneration, thrombosis and late deterioration [7–11]. In a case series of 12 patients, Cardozzo et al. mentioned one patient, who suffered from pseudoaneurysm of the anastomosis between the FV without any long-term follow-up [3]. Valentine et al. reported 2 out of 41 patients, who suffered from anastomotic intimal hyperplasia of the proximal anastomosis causing a stenosis, which could be corrected by intervention [12]. But again, there were no data on the morphometric changes of the anastomosis. To date, few studies have reported long-term outcome results after usage of FV as graft material for reconstruction of the aorto-iliac segment [13]. In particular, there is a lack of essential information about morphometric alterations of the FV at the level of the anastomoses.

### Methods

#### Study population

Between August 2001 and January 2020, 22 Caucasian patients received a reconstruction of the aorto-iliacal segment with the use of the autologous FV at the Medical University of Vienna, Vienna General Hospital. The study has been reviewed and approved by the Ethics Committee of the Medical University of Vienna (EK 20,124/2015), and all study subjects gave written informed consent. In addition, the study adhered to the STROBE criteria and has been registered at researchregistry with the ID researchregistry 5637. Following characteristics and the presence of comorbidities were documented: age, gender, BMI (<19, 20–25, >25 kg/m^2^), peripheral vascular disease and cardiovascular disease, diabetes mellitus type 2, smoking status, coronary artery disease (CAD)[14]] (history of angina pectoris/myocardial infarction (MI), renal insufficiency (glomerular filtration rate < 50 ml/min/1.73m^2^) [15], the presence of peripheral vascular occlusive disease (PAOD, according to Fontaine classification), chronic obstructive pulmonary disease (COPD), cerebrovascular disease, neurological disorders and malignant tumours.

#### Study outcome

We measured morphological changes at the proximal and distal anastomotic sites and graft lumen alterations on 109 follow-up computed tomography angiography (CTA) scans. Graft patency and postoperative 30-day mortality rates were evaluated. The influence of the FV harvesting on venous outflow on the donor leg(s) was examined using measurements of thigh diameters.

#### Follow-up

The vital status of the patients was documented using the mortality database of Statistics Austria on January 1, 2020, and the patients alive at that date was censored.

#### Surgery

Availability, diameter, and morphologic quality of the FV were evaluated preoperatively via duplex ultrasound. A minimum diameter of 6 mm was determined as the essential requirement for use. Femoral veins with chronic occlusion were ruled out. Suitable FV was harvested in 4/22 (18%) uni- or in 18/22 (82%) bilaterally by a longitudinal incision along the medial sulcus of the thigh. All side-branches of the FV were ligated and additionally sewed using 5/0–6/0 prolene. The FV was harvested from its junction with the deep femoral vein including a proximal portion of popliteal vein. An autologous bifurcated graft was created with the FV. After exposition of abdominal aorta and iliac vessels via laparotomy, the infected prosthesis was removed, and the infected tissues debrided. In case of mycotic aortic aneurysms, certainly only the vessel dissection was performed. The aorto-iliac segment was replaced by autologous material. In case of appearance of aorto-enteral fistulae, an omentoplasty was performed for prevention of secondary infection. Microbiological specimen was taken from the infected tissue. According to antibiogram, broad-spectrum antibiotics were given perioperatively, and anticoagulation was performed as required.

#### Morphometric CT angiography analysis

All patients received preoperative and yearly postoperative CTAs (Magnetom Avanto, Siemens Medical Systems, Erlangen, Germany), in total 109 CTAs which were analysed considering the graft patency and morphologic changes of the venous graft. All patients received a CTA scan of the same CTA equipment based on the following settings: Rev. KV 80, iterative reconstruction, patients will receive 40 ml of Iomeron 400 mg/ml contrast agent. Scans of 1 mm slices will cover the entire region of proximal aortic as well as distal iliac or femoral anastomoses. Morphometrical analysis was performed in IMPAX and Syngo Via software to assess the three-dimensional changes of the proximal aortic as well as distal iliac or femoral anastomoses. To evaluate possible postoperative venous or lymphatic outflow problems after FV harvesting, upper thigh and lower thigh diameters were measured by CTA and compared pre- and postoperatively. All measurements were performed by investigators, who were blinded to patients’ data and characteristics. Multiple measurements of the same CTA image were performed and analysed by two experts with an interobserver reproducibility of 0.2 mm and ICC (intraclass correlation coefficient) of 0.999 [16].

#### Statistical analysis

Median (quartile) values were given to describe continuous variables, and absolute numbers and percentages were used to describe categorical variables. Differences in continuous variables of patients between different time points and CTA measurements were tested using the paired two-sample t-test, and non-normally distributed variables were compared by the Wilcoxon rank sum test. Intraclass correlation coefficient with 95% CI was analysed for reliability analysis [16]. Categorical variables were compared by the Chi-square test or the Fisher’s exact test, as appropriate. Correlations of continuous variables were characterized using the Spearman correlation coefficient, and survival rates were calculated according to Kaplan–Meier survival analysis. All *P* values are results of two-sided tests, and *p* values < 0.05 were considered as statistically significant. The SPSS software version 24.0 (IBM corporations Inc. 1989–2016; Amonk, NC, the USA) was used for statistical analyses.

## Results

### Patient’s characteristics

Demographic data and patient’s characteristics (indications for autologous reconstruction were in 10/22 (45%) prosthetic graft infection, in 7/22 (32%) mycotic aneurysms and in 5/22 (23%) aortitis) are summarized in Table 1. There were no significant differences regarding demographic parameters between the three study groups (mycotic graft infection, mycotic aneurysm and aortitis). Common cardiovascular risk factors were in 19/22 (86%) hypertension, in 18/22 (82%) nicotine abuse and in 11/22 (50%) hyperlipidaemia.

**Table 1:** Demographic data of patient’s characteristics (Data are presented as frequencies or median quartiles)

	Mycotic graft infection (*n* = 10)	Mycotic Aneurysm (*n* = 7)	Aortitis (*n* = 5)	Total (*n* = 22)
Age [years], median (quartiles)	63.4 (57.6–67.1)	68 (56.5–74.3)	60.9 (44.8–69.6)	63.4 (56.5–69.9)
Male sex, *N* [%]	8 (80)	5 (71.4)	5 (100)	18 (81.8)
Hypertension, *N* [%]	9 (90)	6 (85.7)	4 (80)	19 (86.4)
Coronary artery disease, *N* [%]	4 (40)	1 (14.3)	2 (40)	7 (31.8)
Smoker, *N* [%]	8 (80)	6 (85.7)	4 (80)	18 (81.8)
Hyperlipidemia, *N* [%]	4 (40)	6 (85.7)	1 (20)	11 (50)
Body mass index, [kg/m^2^] median (quartiles)	23.2 (20.8–27.7)	24.6 (22.4–26.4)	21.1 (20.3–23.4)	23.2 (20.8–27.1)
Diabetes Type II, *N* [%]	1 (10)	1 (14.3)	0 (0)	2 (9.1)
PVD, *N* [%]	7 (70)	0 (0)	1 (20)	8 (36.4)
CVD, *N* [%]	5 (50)	0 (0)	2 (40)	7 (31.8)
Previous MI, *N* [%]	1 (10)	0 (0)	0 (0)	1 (4.5)
Creatinine [mg/dl], median (quartiles)	1.10 (0.90–1.38)	0.92 (0.85–1.13)	0.88 (0.86–0.94)	0.94 (0.86–1.21)
Renal Insufficiency, *N* [%]	5 (59)	2 (28.6)	0 (0)	7 (31.8)
Pulmonary disorder, *N* [%]	6 (60)	2 (28.6)	2 (40)	10 (45.5)
Neurologic disorder, *N* [%]	1 (10)	1 (14.3)	1 (20)	3 (13.6)
Malignant tumour, *N* [%]	3 (30)	0 (0)	0 (0)	3 (13.6)
Previous infection, *N* (%)	1 (10)	4 (57.1)	2 (40)	7 (31.8)

*PVD* peripheral vascular disease, *CVD* cardiovascular disease, *N* number patients,

## Procedure-related details

**Fig. 1:**
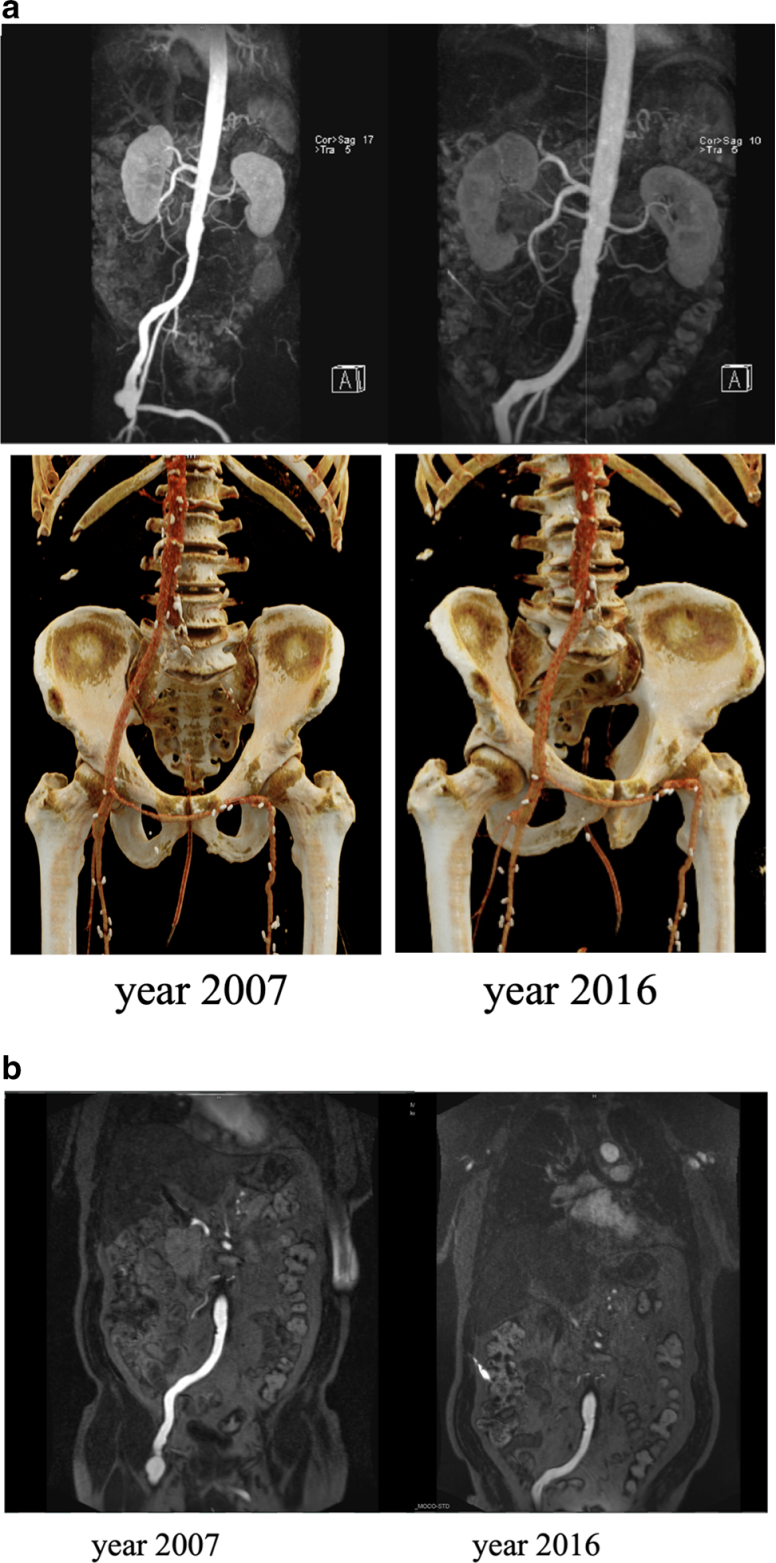
Aorto-iliac reconstruction with extra-anatomic femoro-femoral crossover bypass. Postoperative computed tomography angiography (CTA) scan with three-dimensional reconstruction two weeks after surgery **a** and after 9 years **b**. Proximal as well as distal anastomoses are elongated; however, the bypass is patent

**Fig. 2:**
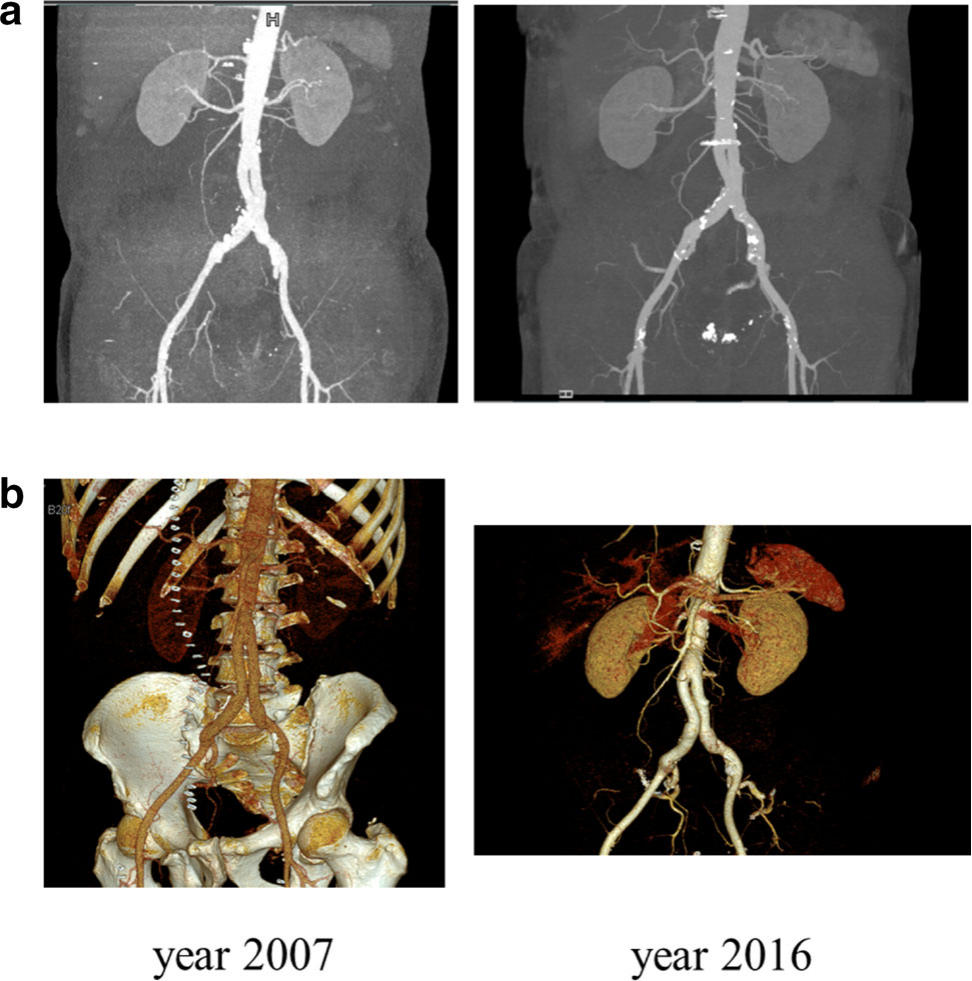
Bifurcational “pantalon” FV graft [17]. Postoperative CTA scan with three-dimensional reconstruction after 1-month **a** and 9 years **b** after surgery. Again, proximal as well as distal anastomoses are significantly elongated; however, the bypass is patent

Elective surgery was performed in 17/22 (77%) cases; emergency intervention was necessary in 5/22 (23%) patients. One patient received sole aorto-iliac reconstruction with extra-anatomic femoro-femoral crossover bypass (Fig. 1 a, b). Bifurcational “pantalon” FV graft was implanted in 17/22 (77%) patients (Fig. 2 a, b) and autologous tube-graft—in 3/22 (14%) patients [17]. In one patient, an extra-anatomical axillary-bifemoral bypass was implanted.

## Microbiological specimen

Microbiologic specimens from the infected tissue were obtained intraoperatively in 20/22 (91%) patients and sent for analyses of bacterial culture and antibiogram (Table 2). In 10/20 (50%) cases, microbial growth was not observed. *Staphylococcus aureus* was most frequently detected in positive bacterial culture specimens in 4/20 (20%) patients. Postoperatively, no reinfection of autologous prosthesis was found.

**Table 2:** Microbiological specimen *n* = 20 (Data are presented as frequencies or median quartiles)

	Total	Graft infection	Mycotic Aneurysm	Aortitis
*No growth*	10 (50%)	4 (20%)	5 (25%)	1 (5%)
*Staphylococcus Aureus*	4 (20%)	2 (10%)	0 (0%)	2 (10%)
*MRSA*	2 (10%)	2 (10%)	0 (0%)	0 (0%)
*Gram-positive Cocci*	1 (5%)	0 (0%)	0 (0%)	1 (5%)
*Enterococci*	1 (5%)	1 (5%)	0 (0%)	0 (0%)
*Aspergillus fumigatus*	1 (5%)	0 (0%)	0 (0%)	1 (5%)
*Salmonella*	1 (5%)	0 (0%)	1 (5%)	0 (0%)

*MRSA* Methicillin-resistant Staphylococcus aureus

## Perioperative complications

Perioperative short- and long-term complications as well as re-interventions were documented in 7/22 (32%) patients. 30-day complications included: seroma formation in 7/22 (32%) patients, ischaemia of the leg requiring thrombectomy in 3/22 (14%) patients, leg oedema without pathological finding in one (5%) patient resolving within 3 months and colon ischaemia in 2/22 (9%) patients. One above-knee-amputation (5%) was performed in the long-term follow-up period because of progression of peripheral artery disease (PAD) without direct correlation to the autologous reconstruction. One patient with PAD stage IIb (Fontaine) received an endovascular stent implantation of the femoral artery 11 months prior to the FV implantation. Forty-nine days after aorto-iliac reconstruction, a possible thrombo-embolic event within the stent in the femoral artery, represented a life-threatening situation for the patient without limb salvage.

## Patency rates

Primary and secondary patency rate was 21/22 (95%). After more than 16 years of follow-up, patency rate was still 95%. The median follow-up was 91.5 months (P_25_;P_75_ = 21;117). The 30-day mortality rate was 3/22 (14%). The 1-year survival rate was 17/22 (77%), and the 5-year survival rate was 9/22 (41%).

## Anastomotic and venous wall thickness measurements

**Fig. 3:**
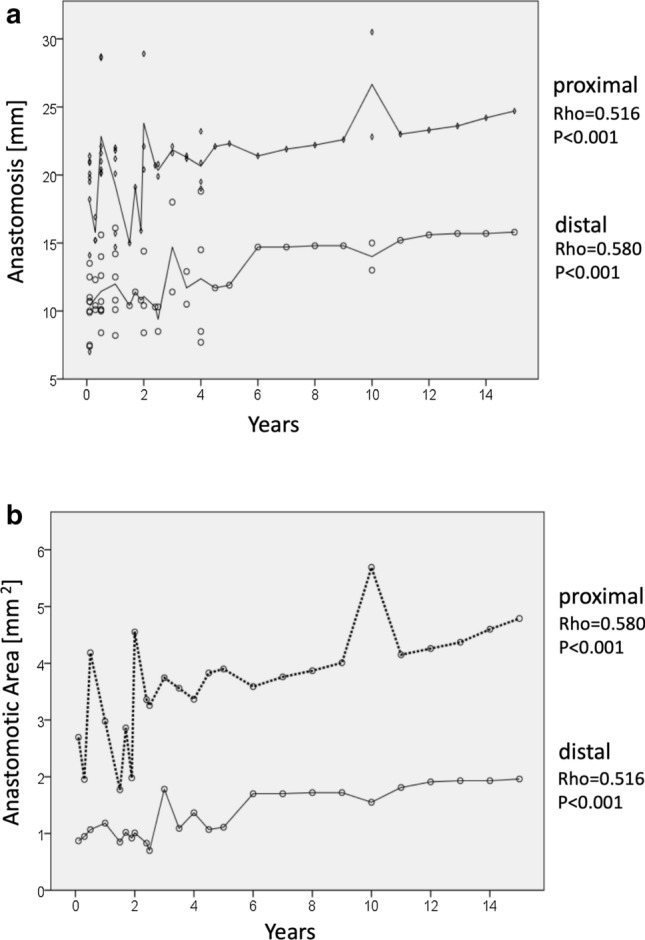
Morphometric analysis of the proximal aortic **a**, distal femoral **b** anastomosis in mm

Cross-sectional diameter of proximal (20.38 ± 3.77 vs 22.04 ± 3.97 mm, *p* = 0.007) and distal anastomoses (13.05 ± 4.23 vs 14.61 ± 5.19, *p* = 0.05) increased significantly (Fig. 3a), as well as proximal and distal anastomotic areas (3.36 ± 1.29 vs 4.32 ± 1.63 mm^2^, *p* = 0.04, and 0.99 ± 0.48 vs 1.25 ± 0.72 mm^2^, *p* = 0.023, respectively) over the observation time (Fig. 3b). The venous wall thickness was significantly reduced at the proximal and distal anastomotic site (1.74 ± 0.46 mm vs 1.24 ± 0.31 mm, *p* = 0.001).

## Examination of venous and lymphatic outflow on the donor legs

The upper thigh diameter differed not significantly before and after harvesting of the FV (161.6 ± 29.1 vs. 178.2 ± 23.3 mm, *p* = 0.326, respectively).

## Discussion

Femoral vein for the surgical reconstruction of infected grafts within the abdominal bifurcation is an already established procedure in selected cases [17, 18]. We show in our tertiary referral hospital up to 16 years of experience with the FV as a proper substitute for aorto-iliac reconstructions in secondary graft infection and mycotic aneurysm with consecutive dilatation of proximal and distal anastomoses and without any signs of secondary rupture or complication on the long term. Our patients were often male with a high degree of hypertension, hyperlipidaemia and nicotine abuse. This outcome strongly corresponds to the typical characteristics and risk factors of patients with atherosclerotic diseases [19–21].

The microbiological cultures revealed *Staphylococcus aureus* as the most frequent pathogen with 20%. This finding is in accordance with other studies examining the microbiology of aortic infections [6, 22, 23]. However, no growth could be shown in 10 out of 20 specimens, which explains the difficulty of identifying the right pathogen and subsequent specific antibiotic treatment. In all cases, surgery could eliminate the source of infection, and no reinfection was documented in our patient cohort. In most cases, reconstruction of the aorto-iliac bifurcation with FV and wide debridement leads to successful avoidance of reinfection [3].

The femoral vein overall patency rate was 95%, even after 16 years of follow-up. Clagett et al. showed a loss of patency in 64% in 22.5 ± 16 months within their patient group treated with the greater saphenous vein —mostly intimal hyperplasia reasoned the avoidance of these small-luminal venous autografts [17]. Excellent patency rates of 91%-100% are published in several studies for the superficial femoral vein, which is in accordance with our data [1, 3, 12, 18, 24].

Considering the extent of surgery by aorto-iliac reconstruction by FV, our 30-day mortality rate was 14%, and 5-year survival rate was 41%. Compared to 49 patients, who received FV reconstruction by Daenens et al., short-term mortality rate was only 8%, and 5-year follow-up was comparable to our study about 40% [18]. Data from the Netherlands showed that 30-day mortality was found to be up to 28% [1], much higher than in our patient group.

Our focus on the morphometric changes of the FV measured by CTA within the prosthesis is unique throughout the literature. Gibbons et al. found 4 out of 10 patients with more than 70% distal anastomotic graft stenosis within 6 months, which could be treated successfully by percutaneous angioplasty. They blamed their anastomotic technique for this result, and this group hypothesized that single spatulation of the vein for anastomosis caused angulation. In case of bi-valving the vein, the venous anastomosis was more symmetrical, and there were no signs of stenosis to be found [25]. Clagett identified one patient, which had a distal femoral artery stenosis after 49 months and one patient with proximal end of FV crossover limb stenosis, which required both thrombectomy and patch correction. Our results are in line with the literature, and venous reconstruction of the aorto-iliac axis does not cause any aneurysmatic or stenosis deterioration of the graft in long-term experience, and short-term defects could only be due to technical issues of the anastomosis [18, 26, 27]. Clagett et al. performed follow-up in FV grafts in 6 and 60 months post-surgery and compared only duplex sonography imaging diameter of the FV, and he could not find any difference ranging from 10.8 ± 1.1 mm to 7.8 ± 1.1 mm. In a subgroup analysis of ten patients, who had serial measurements, initial FV graft diameter was 10.3- + 1.4 mm, and after one year, these ten patients had 8.7 ± 1.6 mm grafts. Ten patients with 10 examinations are only a small sample size. In our analysis, more than 100 CTAs after a mean follow-up of up to 92 months in our group were performed. Secondly, this was only short outcome data, complete data analysis of Clagett et al. showed no significant results after 60 months [26]. Moreover, these are only duplex sonography images, which have a low accuracy compared to CTAs.

In our patient collective, there was no venous outflow problem after resection of the FV. Comparing morphometric analysis diameter of the legs before and after surgery, there was no significant difference in our study. There are studies, which postulate postoperative lymphatic swelling; however, these studies did not undergo objective measurement by CTA of diameter of upper and lower legs and only describe lymphatic swelling [26, 28]. Furthermore, postoperative swelling could be caused by chronic infection and short-term follow-up in these studies cannot sufficiently analyse this problem. Harvesting the FV is well tolerated due to extensive collateral venous network in the leg and causes only minimal venous morbidity [29–32]. Alternatives like the saphenous vein show failure rates up to 64% compared with 0% for superficial femoral vein reconstruction and should therefore be only second choice [17].

Since the inclusion criteria for this study are infection of pre-implanted prostheses and/or mycotic abdominal aortic aneurysm as well as aortitis, the sample size of this study is rather small; however, more than 100 CTAs have been performed. Prosthesis infection is resistant to conservative treatment and occurs seldom, and therefore, studies with large patient populations are not to be expected. In the known literature, we did not find long-term data concerning morphometric analysis using CTAs [24, 26, 27].

There are several limitations, which must be addressed. First, it should be noted that the data for this study were collected retrospectively (therefore no specific prospective study protocol). Resulting, no possibility to study factors associated with dilatation of the anastomosis due to small sample size and no possibility to adjust for confounding is given. However, reliable judgments about the external validity are difficult. The population characteristics of this study cannot exclude a sampling bias or influencing factors related to the study period.

### Conclusion

This long-term CTA follow-up study showed that the FV wall becomes thinner at the anastomotic site and the anastomoses dilate with time without rupture. The FV is a durable conductor after replacement of the aorto-iliac segment due to aortic infection. Further CTA studies from more centres are warranted to evaluate the risk of vein rupture.
